# Gut-Brain Endocrine Axes in Weight Regulation and Obesity Pharmacotherapy

**DOI:** 10.3390/jcm3030763

**Published:** 2014-07-15

**Authors:** Dante J. Merlino, Erik S. Blomain, Amanda S. Aing, Scott A. Waldman

**Affiliations:** Department of Pharmacology and Experimental Therapeutics, Thomas Jefferson University, 1020 Locust Street, JAH 368, Philadelphia, PA 19107, USA; E-Mails: dante.merlino@jefferson.edu (D.J.M.); erik.blomain@jefferson.edu (E.S.B.); amanda.aing@jefferson.edu (A.S.A.)

**Keywords:** obesity, satiety, hypothalamus, gut-brain endocrine axis, pharmacotherapy

## Abstract

In recent years, the obesity epidemic has developed into a major health crisis both in the United States as well as throughout the developed world. With current treatments limited to expensive, high-risk surgery and minimally efficacious pharmacotherapy, new therapeutic options are urgently needed to combat this alarming trend. This review focuses on the endogenous gut-brain signaling axes that regulate appetite under physiological conditions, and discusses their clinical relevance by summarizing the clinical and preclinical studies that have investigated manipulation of these pathways to treat obesity.

## 1. Introduction

Over the last 50 years, rates of obesity have escalated into a global pandemic [1]. In the United States, two-thirds of the adult population is classified as overweight (BMI > 25 kg/m^2^), with half of that population classified as obese (BMI > 30 kg/m^2^) [2]. Obesity is associated with co-morbidities that reduce life expectancy and incur substantial treatment costs, including breast and colon cancer, depression, type 2 diabetes, heart disease, hypertension, infertility, liver disease, sleep apnea, osteoarthritis, and stroke [3]. With trends in this epidemic worsening, it is clear that safe and effective treatments are urgently needed. One therapeutic approach that is rapidly gaining momentum is the manipulation of endogenous gut-brain endocrine axes responsible for maintaining homeostasis between energy intake and expenditure.

## 2. Current Treatment Options

While therapeutic and preventative modalities exist to combat obesity, success thus far has been limited. Lifestyle changes, a strategy which incorporates improvements in diet and exercise, have been shown to reduce weight [3] and minimize co-morbid conditions [4,5] but, unfortunately, these treatment modalities are often associated with poor patient compliance and thus are rarely maintained [6,7].

To date, bariatric surgery (gastric bypass, gastric banding, and sleeve gastrectomy), is the most successful anti-obesity treatment in terms of sustained weight loss and amelioration of co-morbidities [8,9]. While the degree of success varies by procedure, these surgical interventions typically result in sustained excess weight loss of 40%–60% [10,11]. However, like all major surgical interventions these procedures can result in severe and even life-threatening complications, including infection, gastric perforation, incisional hernias, and nutritional deficiencies. Serious complications occur in 3%–4% of patients, with reoperation required 1% of the time [12]. A prospective, randomized clinical trial comparing gastric bypass and gastric banding demonstrated treatment failure (defined as excess weight loss of less than 20%) in 16.7% of patients treated with gastric banding [13], with long-term data suggesting failure rates as high as 40% seven years after a gastric band procedure [14]. Due to the cost and high complication rates, these treatments are limited to the morbidly obese (BMI > 40 kg/m^2^) and those suffering from grave co-morbidities. Unfortunately, these highest-risk patients are also often poor candidates for surgery and may go untreated, as demonstrated by the fact that only 1% of patients with morbid obesity undergo bariatric surgery in a given year [15].

Given these limitations of lifestyle modifications and surgery, the development of anti-obesity pharmacotherapeutics will be vital to the prevention and treatment of obesity. Thus far, ongoing safety concerns have limited the implementation of these pharmacological treatments for obesity. Until 2012, only one drug, orlistat (Alli^®^, GlaxoSmithKline, Brentford, England), had been approved by the United States Food and Drug Administration (FDA) as a long-term weight loss drug [16,17]. After previous rejections in 2010, phentermine/topiramate (Qsymia™, Vivus, Mountain View, CA, USA) and lorcaserin (Belviq^®^, Arena Pharmaceuticals, San Diego, CA, USA) were finally approved by the FDA for the treatment of obesity in 2012. Despite some optimism over these new therapies, concerns persist regarding the safety and efficacy of available treatments and demand further research aimed towards developing new therapeutic options for the treatment of obesity.

In an effort to develop new anti-obesity therapeutic agents, significant work has focused on elucidating the pathways which naturally mediate appetite and energy balance. Such research initially emphasized endocannabinoid and monoamine signaling in the brain, resulting in the development of rimonabant, a cannabinoid-1 receptor antagonist, and sibutramine, a selective serotonin/norepinephrine reuptake inhibitor. Unfortunately, these drugs were riddled with adverse events, including depression [18] and increased risk of cardiovascular events [19], respectively. These events resulted in withdrawal of these compounds from the market shortly after their approval. More recently, focus on the hormonal endocrine regulation of appetite has led to several promising candidates for the safe and effective treatment of obesity. Investigation of these signaling pathways in the central nervous system, as well as their manipulation with hormonal analogs or antagonists will be the focus of this review.

## 3. The Hypothalamus is the Primary Site of Central Appetite Regulation

The hypothalamus is the center of appetite regulation, integrating neural and endocrine signaling so that food intake matches energy demand [4,20,21]. Distinct hypothalamic neurons utilize specific neuropeptides and signaling pathways to stimulate feelings of hunger or satiety. Understanding the regulation of these nuclei and their roles in homeostatic signaling may improve understanding of the pathogenesis of obesity, as well as potential targets for therapeutic intervention in obese patients.

### 3.1. The Arcuate Nucleus

The arcuate nucleus (ARC) is one of the central sites of appetite regulation in the hypothalamus. This periventricular organ, located ventrolateral to the third ventricle, receives its blood supply via fenestrated capillaries, allowing it access to endocrine signals from other areas of the body [20,22]. Neurons in the ARC with receptors to these endocrine signals project to other areas of the brain, including other nuclei in the hypothalamus, to control feelings of hunger and satiety. Anorexigenic neurons, stimulated by endocrine signals following ingestion of a meal, generate impulses that suppress appetite and induce satiety. These neurons mediate their effect through the production, post-translational modification, and release of two neuropeptides, pro-opiomelanocortin (POMC) and cocaine-and amphetamine-regulated transcript (CART) to second-order neurons in the brain. Conversely, in fasting conditions, orexigenic neurons in the ARC utilize agouti-related peptide (AgRP) and neuropeptide Y (NPY) to induce feelings of hunger [20,21,23]. The integration of these signals is a critical determinant of ultimate caloric intake.

### 3.2. Pro-Opiomelanocortin

Pro-opiomelanocortin (POMC) is a 241-amino acid precursor peptide that can be selectively cleaved to generate eight unique neuropeptides. The neuropeptide most commonly associated with appetite regulation is α-melanocyte-stimulating hormone (α-MSH), which binds to and activates an α-MSH receptor, MC3R or MC4R, present on the surface of second-order neurons [24]. Food intake is reduced when agonists to these G protein-coupled receptor are administered intracerebroventricularly (ICV); similarly, ICV administration of MC3R or MC4R antagonists results in reduced caloric intake in rodents [25]. The anorexigenic properties of α-MSH signaling are also demonstrated by hyperphagia-induced obesity that develops following targeted destruction of MC4R [26], as well as the association between late-onset obesity and polymorphisms of the MC4R in human populations [27]. Recent studies suggest that impaired α-MSH signaling may also play a role in the pathogenesis of obesity: in rodents, obesogenic diets cause endoplasmic reticular (ER) stress in hypothalamic neurons, resulting in impaired POMC post-translational processing and reduced postprandial α-MSH release [28].

Initial clinical investigation of α-MSH-R agonists generated discouraging results due to their significant adverse event profile. Intravenous (IV) administration of one such agonist, LY2112688, resulted in muscle stiffness and penile erection, as well as a dose-dependent increase in blood pressure and heart rate in overweight and obese human subjects [29,30]. However, a newly developed, highly selective MC4R agonist, RM-493 (formerly BIM-22493) (Rhythm Pharmaceuticals, Inc., Boston, MA, USA), did not demonstrate these adverse effects when administered to diet-induced obese (DIO) rhesus macaques. Minipump infusion of 0.5 mg/kg/day of RM-493 resulted in sustained weight loss that continued throughout the course of the treatment; after eight weeks of treatment, DIO macaques lost an average of 10% of their body weight, with 75% of treated animals continuing to lose weight for two weeks after cessation of treatment. Interestingly, the weight loss appeared to be initiated by a transient reduction in caloric intake, and sustained by a steady increase in daytime activity, which nearly doubled by the termination of treatment after eight weeks. RM-493 also reduced fat mass an average of 18.8% and improved insulin sensitivity in DIO macaques [31]. A phase II clinical trial investigating weight loss induced by RM-493 at a dose of 1 mg/day via subcutaneous infusion for 90 days is currently in progress [32]. In addition, a separate MC4R agonist, AZD2820 (Astra Zeneca, London, England) helped DIO mice maintain weight loss initially achieved by caloric restriction [33]. These early findings suggest that α-MSH-R agonists may eventually play an important role in the treatment of obesity.

### 3.3. Cocaine-and Amphetamine-Regulated Transcript

ARC neurons that produce POMC co-express another anorexigenic neuropeptide, cocaine-and amphetamine-regulated transcript (CART), that induces satiety when presented to second-order neurons in the hypothalamus. Like POMC, ICV administration of recombinant CART reduces feeding in rodents, and antagonism of CART signaling via ICV administration of CART antiserum increases caloric intake [34]. However, direct stereotactic injection of CART into the ARC has been shown to increase feeding in rats, suggesting that CART induces different downstream effects in different areas of the hypothalamus [35]. This ambiguity, coupled with the fact that the CART receptor has yet to be identified has quelled interest in clinical translation of the CART pathway to treat obesity.

### 3.4. Agouti-Related Peptide

Agouti-related peptide (AgRP) is a neuropeptide expressed in orexigenic neurons of the ARC [36]. The peptide, a potent selective antagonist of the MC3R and MC4R, induces hunger by inhibiting the downstream signaling caused by the release of α-MSH on second-order neurons in the hypothalamus [37]. As a result, transgenic mice overexpressing AgRP exhibit a hyperphagic, obese phenotype, and mice administered AgRP ICV exhibit increased food intake [38]. In 2009 a specific AgRP inhibitor (TTP-435, TransTech Pharma, High Point, NC, USA) entered phase II clinical trials, but the results of this trial were not released [39,40].

### 3.5. Neuropeptide Y

Neuropeptide Y (NPY), expressed by orexigenic neurons in the ARC, is a potent stimulator of appetite [41]. As a member of the pancreatic polypeptide family, NPY is capable of binding to six GPCRs (Y1–Y6) [42]; early studies indicate that activation of the Y1 and Y5 receptors stimulates appetite [43], while Y2 and Y4 act presynaptically to inhibit further NPY release [44]. Overexpression of NPY in the dorsomedial hypothalamus, a major site of signaling from ARC neuronal projections, induces hyperphagia and obesity in rats [45]. Like POMC, NPY signaling may be influenced by ER stress induced by obesogenic diet, and may play a role in the development of obesity. In cultures of mouse hypothalamic nuclei, induction of ER stress dramatically increased mRNA expression of NPY [46].

Studies investigating the utility of NPY receptor antagonists as potential therapeutic options for the obesity are ongoing. Administration of an 800 mg/day of an oral Y5-receptor antagonist, velneperit (formerly S-2367, Shionogi USA, Inc., Florham Park, NJ, USA) resulted in a placebo-adjusted weight loss of 3% of baseline weight, with 35% of patients losing at least 5% of their baseline weight [29]. In 2011, a phase II clinical trial was completed that investigated the weight loss benefits of velneperit as monotherapy or in conjunction with orlistat, but to this point no positive results are available [47].

## 4. Endocrine Regulation of Satiety and Energy Homeostasis

### 4.1. Cholecystokinin

Cholecystokinin (CCK) is secreted by primarily by I-cells in the proximal intestinal mucosa upon exposure to fatty acids and proteins, and has also been characterized as a neurotransmitter in the central nervous system [48,49]. CCK binds to one of two G protein-coupled receptors, CCK-A or CCKB. Administration of selective agonists and antagonists has demonstrated that CCK mediates satiety by binding to the CCK-A receptor [50,51,52,53]. However, this effect does not appear to mediate long-term changes in caloric intake. While rats administered CCK do show reduced caloric intake during a meal, a compensatory increase in feeding frequency abrogates any effects that this would have on caloric intake [54]. Furthermore, tachyphylaxis to CCK tolerance develops rapidly following exogenous administration of CCK [55], and DIO rats exhibit reduced sensitivity to CCK [56]. These results, coupled with the preclinical finding that CCK administration increases the incidence of pancreatitis in rodents [57], suggest that CCK agonists are not appealing candidates for monotherapy of obesity, although their efficacy in combined treatments cannot be ruled out.

### 4.2. Glucagon-Like Peptide 1 Agonists

Glucagon-like peptide 1 (GLP-1) analogs have recently received much attention as a potential treatment for obese patients with type 2 diabetes (T2DM), and the early success of GLP-1 analogs suggests that the role of this gut peptide in the treatment of diabetes and obesity is set to expand. Four GLP-1 receptor agonists (GLP-1 RA) have been approved for the treatment of T2DM in the past 10 years, with others in clinical or preclinical testing. In addition to the weight loss benefits that GLP-1 RAs provide, they improve glycemic control [58], systolic and diastolic blood pressure [59], and cholesterol plasma levels [60], without significantly increasing risk of hypoglycemia.

There are two biologically active forms of GLP-1, GLP-1(7-37) and GLP-1(7-36)NH_2_, both derived from the transcriptional product of the preproglucagon gene. It is produced in the L-cells of the ileum and proximal colon and is secreted postprandially [61]. As an incretin, GLP-1 stimulates the release of insulin by pancreatic β-cells; it also delays gastric emptying, suppresses glucagon secretion, and plays a central role in appetite regulation [62]. The GLP-1 receptor (GLP-1R) is not limited to the pancreas; this GPCR is expressed throughout the body, including the lung, kidney, and central and peripheral nervous system [61]. Because this receptor can couple to Gα_s_, Gα_q_, Gα_i_, and Gα_o_ [63,64], the signaling cascade it initiates varies widely depending on the cell in which it is expressed. In the hypothalamus, the GLP-1R is expressed in the ARC as well as the paraventricular nucleus [65].

Studies have shown that both ICV and peripheral administration of GLP-1 rapidly induces satiety in rodents, and continuous exposure results in long-term weight loss. This response appears to be centrally mediated both by ARC signaling as well as vagal signaling. Destruction of either the ARC or subdiaphragmatic vagotomy abrogates the anorectic effect of GLP-1 administration [66,67]. GLP-1 is rapidly degraded in the bloodstream due to its degradation by dipeptidyl peptidase IV (DPP-IV), resulting in a half-life of only two minutes [68]. Recent discoveries of the DPP-IV-resistant GLP-1 RA and modifications to the biological GLP-1 peptide have dramatically improved this half-life, improving its utility as a therapeutic.

Exendin-4, isolated from the venom of the gila monster in 1992, shares only 53% of sequence identity with the biologically active GLP-1 and as a result is resistant to DPP-IV cleavage. Despite this, exendin-4 remains a potent GLP-1 RA [69]. Exenatide BID (Amylin, now Bristol-Myers Squibb, New York, NY, USA), a synthetic form of exendin-4 with a terminal half-life of ~2.4 h, in 2005 was the first GLP-1 RA approved for the treatment of type 2 diabetes. Like all GLP-1 RA currently approved for patient use, it is administered via subcutaneous injection, but because of its relatively short half-life, is dosed twice daily (10 μg). An 82-week trial of combination exenatide and metformin resulted in an average weight loss of 4.4 kg [70]. Another synthetic analog of exendin-4, lixisenatide (Sanofi/Zealand), has a mean terminal half-life of 3 h. Lixisenatide has a milder adverse effect profile than exenatide, but also results in milder weight loss [71]. In February 2013, lixisenatide was approved for the treatment of T2DM by the European Commission, but in 2013 Sanofi elected to delay the FDA approval process pending the results of an internal cardiovascular risk study due in 2015 [72].

Liraglutide (Victoza^®^ Novo Nordisk, Bagsværd, Denmark), shares 97% homology to native GLP-1 (7-37), but is conjugated to palmitic acid [73]. This modification allows for the peptide to be gradually released from the site of injection. Once it enters the bloodstream, the peptide binds extensively to albumin, stabilizing it against degradation [74]. As a result, liraglutide possesses one of the longest terminal half-lives of currently approved GLP-1 RA, at 11–13 h. Furthermore, liraglutide has been demonstrated to safely induce weight loss in obese non-diabetic patients. In a clinical trial investigating the effects of liraglutide on non-diabetic obese individuals, treated patients exhibited a dose-dependent weight loss of 5.0–7.2 kg after 20 weeks of treatment. Furthermore, three-fourths of patients administered the maximum 3.0 mg dose of liraglutide exhibited a weight loss of at least 5% of their baseline weight, with 28% achieving a reduction of 10% or more of their baseline weight [75]. Despite these findings, liraglutide is not marketed as a weight-loss drug, and is approved only for the treatment of type 2 diabetes.

#### GLP-1 Receptor Agonists with Extended Duration

Research is currently underway to modify current GLP-1 RA to reduce the number of required injections in order to improve patient compliance. Taspoglutide, a GLP-1 RA developed by Roche (Basel, Switzerland), which requires dosing only once a week, improved glycemic control and induced weight loss comparable to exenatide. However, significant gastrointestinal side effects, injection site reactions, and systemic allergic reactions caused 34% of patients to discontinue treatment. As a result, Roche terminated the drug’s development [76,77].

Another weekly dosing formulation of GLP-1 RA, exenatide-LAR (Bydureon^®^ (Bristol-Myers Squibb)), is a formulation of exenatide trapped within polymeric microspheres. Gradual breakdown of the microspheres causes a sustained release of exenatide, resulting in a terminal half-life of 5–6 days. Head-to-head comparisons of exenatide LAR to exenatide BID showed comparable weight loss (3.6 *vs*. 3.7 kg) [78], although a separate comparison of exenatide LAR to liraglutide demonstrated increased weight loss in the liraglutide group (3.6 *vs*. 2.7 kg after 26 weeks of treatment). A major disadvantage of exenatide LAR is that the viscous nature of the polymeric suspension requires subcutaneous administration with a 23 gauge needle, which is considerably larger than the needles used to inject other peptide agents [69]. Despite these issues, the FDA approved exenatide LAR for the treatment of T2DM in 2012 [79].

Semaglutide (Novo Nordisk) is a modified form of liraglutide with a plasma half-life of ~160 h, allowing it to be dosed once weekly. A phase II clinical trial demonstrated a placebo-adjusted weight loss of up to 3.6 kg following a 12-week treatment period [80]. It is currently in phase III clinical development for the treatment of T2DM, and phase I efficacy trials of an oral formulation of semaglutide are currently in development [81].

Adverse effects of GLP-1 RA are typically limited to gastrointestinal discomfort, most commonly nausea and vomiting. Fortunately, these effects have mostly been transient can be limited by avoiding rapid dose escalation. Beyond these minor concerns, several cases of acute pancreatitis have been reported in postmarketing surveillance studies, although these cases have not definitively been attributed to GLP-1 RA therapy. In addition, liraglutide administration has been shown to increase the risk of malignant thyroid C-cell carcinomas in rodents. In 2011, in response to these troubling findings as well as the possible risk of acute pancreatitis, the FDA required that Novo Nordisk describe these risks to all potential prescribers. Additionally, liraglutide was not recommended as a first-line therapy for patients with diabetes because of these risks [82].

The potential adverse effects associated with certain formulations of GLP-1 RA may limit their clinical utility, and continued surveillance will ultimately determine whether these drugs are safe enough to be used widely in the treatment of diabetes and obesity. Barring any safety concerns, efficacy studies strongly support the therapeutic potential of GLP-1 receptor agonists both as monotherapy and combination therapy for the treatment of diabetes and obesity. Research to develop longer-lasting or oral formulations of these drugs may even further improve their utility in the treatment of these chronic diseases.

### 4.3. Oxyntomodulin

Similar to GLP-1, oxyntomodulin (OXM) is an alternative cleavage product of the pro-peptide preproglucagon, is produced in L cells of the intestinal mucosa, and is released post-prandially. This 37-amino acid peptide is comprised of the 29-amino acid sequence of glucagon, with eight additional amino acids on its carboxy-terminus. OXM acts as a dual agonist to both GLP-1 and glucagon receptors. This property appears to both reduce appetite while simultaneously increasing energy expenditure [83]. The central anorectic effects of OXM appear to be mediated by GLP-1 receptor signaling in the ARC. Like GLP-1, peripheral administration of OXM increases c-fos expression in the ARC and reduces caloric intake in wild-type mice, but not in *Glp1r^−/–^* mice [84,85]. Three-times-daily subcutaneous injection of OXM for four weeks reduced body weight by a placebo-adjusted average of 1.7 kg in human subjects [83], and a separate study demonstrated that OXM administration both suppressed appetite and increased energy expenditure in patients [83].

Currently, OXM’s therapeutic potential is limited by its short plasma half-life, but, as illustrated by the development of long-lasting GLP-1 RAs, multiple potential strategies exist to overcome this barrier. To this end, in 2012, Zealand Pharmaceuticals began phase I development of ZP2929, a once-daily GLP-1/glucagon dual receptor agonist for the treatment of diabetes and/or obesity. An update on the clinical development of this drug is expected in early 2014 [86].

In April 2013, Transition Therapeutics announced the results of a proof-of-concept study with their once-weekly GLP-1/glucagon dual receptor agonist TT-401. Five-week treatment with TT-401 in obese patients (both with and without diabetes) resulted in statistically significant weight loss in both cohorts, with diabetic patients showing improved glycemic control. Adverse effects tended to be mild, with some patients in the highest dose regimens experiencing nausea and vomiting [87]. In June 2013, Eli Lilly paid Transition therapeutics $7 million to assume all development and commercialization rights to TT-401, and a phase II clinical trial is currently in development [88].

Early results indicating the weight loss and glycemic benefits of dual GLP-1/glucagon receptor agonists such as OXM have initiated the development of a promising new class of drugs for the treatment of obesity. As development of these drugs continues, their safety and efficacy profiles will ultimately determine their role in the pharmacotherapy of diabetes and obesity.

### 4.4. Ghrelin

Ghrelin has the unique distinction of being the only known orexigenic hormone in circulation. Ghrelin is a 28-amino acid peptide hormone originating primarily from the stomach [89]. Interestingly, ghrelin was shown to induce secretion of growth hormone (GH); although the physiological relevance of this secretion is unclear, the receptor that ghrelin binds was consequently named the GH secretagogue receptor (GHS-R) [89,90]. Ghrelin induces feeding and weight gain in both mice and humans [91,92,93]. Obese patients express low levels of ghrelin, while anorexic patients exhibit high levels of the hormone [94,95]. Moreover, changes in body weight seem to modulate ghrelin levels, which fluctuate to oppose changes in body weight [96,97]. These findings suggest that ghrelin may function adaptively to assist in long-term weight maintenance.

Ghrelin induces feeding signals in the brain through several mechanisms. The best characterized of these CNS pathways involves activation of GHSR1a receptors in the arcuate nucleus of the hypothalamus, where ghrelin is believed to activate NPY/AgRP neurons to induce feeding [98,99]. In addition to this canonical pathway, these receptors have also been found in other CNS regions, including other hypothalamic nuclei, the pituitary gland and the hippocampus [98,99]. Importantly, injection of ghrelin directly into these regions also induced feeding, suggesting a multifocal paradigm of action [100,101]. In addition to CNS functions, vagal stimulation is important to the ghrelin response, as vagotomized mice lose their responsiveness to ICV or peripheral administration of ghrelin [102].

Although it is an orexigenic hormone, ghrelin is actually reduced in obesity, confounding its potential clinical utility in battling obesity. Despite this issue, ghrelin has been targeted in the past with a goal of inhibiting the pathway to reduce caloric intake. A vaccine, CYT009-GhrQb (Cytos Biotechnology, Schlieren, Switzerland), was used to exploit this strategy in clinical trials [103]. Development of the product was later discontinued after patients exhibited minimal weight loss despite strong immune responses from the vaccine. Although there were no side effects associated with inhibition, the lack of efficacy introduced doubt over ghrelin’s therapeutic potential in obesity. Casting light on this failure, a more recent study identified endogenous antibodies against ghrelin in obese mice and humans, and showed a role for these antibodies in stabilizing ghrelin. They further demonstrated that these ghrelin-stabilizing antibodies actually increased feeding in obesity. Therefore, immunotherapies designed to raise antibodies against ghrelin may actually exacerbate obesity [104].

Another clinical strategy utilized an RNA Spiegelmer, NOX-B11 (Noxxon Pharma Ag, Berlin, Germany), which binds to and inactivates ghrelin. While this treatment did block the effects of exogenous ghrelin administration [105], rats treated with NOX-B11 alone did not exhibit changes in feeding [106]. Further agents, classed as ghrelin antagonists (Elixir Pharmaceuticals/Novartis and AEterna Zentaris (AEZS-123)), are still in preclinical studies [94,107]. More recent work has identified ghrelin *O*-acyltransferase (GOAT), an enzyme that modifies ghrelin and facilitates receptor binding, and this is viewed as an intriguing target that induces weight loss in preclinical studies [108,109]. Nonetheless, the clinical future of the ghrelin pathway in treating obesity remains uncertain.

### 4.5. Peptide YY

Peptide YY (PYY) is a peptide hormone in the pancreatic polypeptide family, and is secreted from L cells in the intestine [110]. Two isoforms of this hormone exist in humans: PYY_1-36_ and PYY_3-36_, the latter of which is the primary form found in circulation [111]. Both of these isoforms activate the GPCRs Y1–Y6 to activate their downstream effects. Canonically, these hormones have been shown to influence GI motility, as well as to reduce pancreatic and gastric secretion. Interestingly, more recent work also identified a pivotal role for PYY in central regulation of energy balance through the activity of the Y2 receptor in the central nervous system [42,112,113,114]. Importantly, these studies showed that PYY levels increase markedly postprandially, reflecting a strongly anorexigenic effect. Given PYY’s anorexigenic functions, it is not surprising that multiple studies have shown dysfunctional PYY signaling in obesity. This dysfunction was secondary to decreased levels of PYY, and this decrease is reversible, with obese individuals regaining normal circulating levels following bariatric surgery [115].

Centrally, PYY canonically exerts its anorexigenic effects by binding to Y2R receptors in the arcuate nucleus of the hypothalamus as well as regions of the brainstem [114,116]. However, more recent evidence supports a more elegant view of PYY signaling in the brain. PYY administration was shown in neuroimaging studies to induce signaling in many additional areas of the brain [117]. These effects work in tandem to modulate a switch from homeostatic feeding signals to hedonic pathways, largely in the orbitofrontal cortex (OFC) [118,119]. This switch in effect makes food less rewarding, explaining in part the reduced caloric intake following PYY administration.

Given the established role for taste in initiating hedonic food reward mechanisms, it is therefore not surprising that an emerging body of evidence suggests that food taste and food preference influences PYY levels, and that food preference is in turn modulated by PYY, suggesting a possible feedback loop [120,121,122]. Further work is needed, but these findings are potentially important for obesity and related sequelae, which are affected not only by simple caloric intake but also by food preference and macronutrient composition of the diet.

Since PYY dysfunction in obesity reflects hormone deficiency rather than hormone resistance, PYY hormone supplementation is viewed as a potential anti-obesity agent. Clinical studies involving PYY were initially promising, with infusion studies demonstrating that PYY reduced caloric intake and appetite in both obese and non-obese individuals [123,124]. Since infusion is not a feasible clinical mode of administration, later studies were conducted via nasal spray vehicle (Merck Nastech, WA, USA). Unfortunately, this intervention did not yield clinically significant decreases in caloric intake in a randomized clinical trial [125]. Additionally, a high percentage (>50%) of patients withdrew from the study after experiencing side effects which included nausea, vomiting and abdominal discomfort [125]. These effects were dose dependent and possibly due to the bolus dosing platform of the nasal spray. Other clinical routes of administration, such as combinatorial therapies or monotherapies formulated for delayed release to mimic postprandial increases, have thus far not come to fruition.

### 4.6. Pancreatic Polypeptide

Pancreatic polypeptide (PP) is similar in structure to PYY and exerts its effects by similarly binding to GPCRs, notably Y4 and Y5 [126]. PP is predominantly expressed in the pancreas, but can also be found at lower levels throughout the GI tract and in the circulation [127]. Similar to PYY, PP levels rise sharply postprandially, and studies in mice involving injection or overexpression of PP have identified hypophagic effects of PYY [127,128,129]. Importantly, basal and postprandial PP levels are blunted in obese mice and humans, and this observation has been implicated as possibly causing obesity in ob/ob leptin knockout mice [130,131]. In support of this hypothesis, exogenous administration of PP has been shown to reduce caloric in ob/ob mice [130]. Following weight loss, PP levels generally return to levels observed in lean individuals [131].

Peripheral PP acts on the central nervous system through vagal stimulation which allows for downstream accumulation of the anorexigenic neuropeptide urocortin, as well as reduction of orexigenic neuropeptides including NPY and orexin in the hypothalamus [128]. Additionally, PP has been directly localized to the brain, where it has been shown to bind to Y4 receptors in the area postrema, an area highly involved in processing vagal inputs [132,133]. Underscoring the importance of this pathway, PP does not reduce caloric intake in vagotomized mice [128].

Similar to PYY, PP dysfunction in obesity reflects a hormone deficiency. Therefore, exogenous PP administration has been explored as a form of hormone replacement therapy. In human studies, infusion of PP was sufficient to significantly reduce caloric intake at a buffet-style meal [134]. Although no longer-term trials have been conducted for PP in humans, only mild adverse events have been documented. The satiety response was durable up to 24 h, reflecting the comparatively long half-life of PP compared to other satiety hormones [134,135].

Taking advantage of these favorable characteristics, exogenous analogues of this family of peptides are being investigated clinically to mimic PP activity as an agonist for Y2 and Y4 receptors (Obinepitide, 7TM Pharma, Lyngby, Denmark), but as is the case for PYY administration, obinepitide administration caused nausea in humans [136,137,138]. Members of this family of peptides are structurally similar to one another, sharing a PP-fold structure [139]. Despite these similarities, recent work has shown additive effects of co-administration of PYY and PP, with each peptide activating different pathways in the hypothalamus [140]. This suggests distinct mechanisms of action among members of this family and is consistent with the differential tissue distribution of expression of these peptides. More work is needed to identify these functional differences in order to elucidate the molecular events underlying the satiety effects of PP and PYY as well as the adverse events seen following administration. Therefore, despite preliminary work, the long-term clinical outlook of this hormone replacement therapy is still unclear.

### 4.7. Amylin

Amylin, or islet amyloid polypeptide (IAPP), is a peptide hormone which is synthesized and secreted by pancreatic β cells along with insulin [141]. Like insulin, fasting plasma levels of amylin are low and rise considerably postprandially [142]. Amylin works in synergy with insulin to regulate postprandial levels of glucose by enhancing hepatic glycogen synthesis and inhibiting glucagon release from pancreatic α-cells [143].

Along with glucose homeostasis, amylin plays a neuroendocrine role in inducing satiety. This response is believed to be due, at least in part, to amylin’s ability to slow gastric emptying [144]. In addition, ICV administration of amylin in rats reduces food intake in both genetically obese and lean mice [143,145]. Pharmacologic inhibition of amylin signaling via administration of an amylin receptor antagonist increases rodent feeding and fat deposition [146]. Despite this work, studies of transgenic amylin-deficient mice have generated inconclusive results, with some demonstrating an obese phenotype [147,148], while others have found no difference in food intake or weight [149,150,151]. The observed lack of a weight difference between wild-type and amylin deficient mice has thus far been attributed to other mechanisms in the regulation of food intake that compensate for the absence of amylin [151].

Structurally, amylin is similar to a family of peptides that includes calcitonin gene-related peptide (CGRP), calcitonin and adrenomedullin [152,153]. Amylin-specific receptors form from the coexpression of calcitonin receptors and receptor activity-modifying proteins (RAMPs) [154,155]. These receptors are localized to specific areas of the brain, including the area postrema [156]. Vagotomy or lesioning of the area postrema/nucleus tractus solitarius weaken amylin’s effect on satiety and gastric emptying, suggesting that these satiety effects are dependent on vagal signaling [157,158,159].

Since human amylin has a propensity to form insoluble aggregates, Amylin Pharmaceuticals (now Bristol-Myers Squibb, CA, USA) grafted residues from rat amylin, which is not amyloidogenic, into the human amylin sequence. This process resulted in a clinically tractable synthetic amylin analogue called pramlintide, which is both stable and soluble [160]. Studies have demonstrated pramlintide as having a similar pharmacokinetic and pharmacodynamic profile to endogenous amylin and is indicated as an adjunct therapy for type 1 and 2 diabetes, where prandial insulin does not achieve optimal therapy [161].

Randomized clinical trials (RCTs) of pramlintide have focused primarily on the treatment of either type 1 or type 2 diabetes, with weight only studied as a secondary outcome. Each of three different RCTs of type 1 diabetic patients reported that subjects receiving pramlintide exhibited a modest weight loss ranging from 0.4 to 1.3 kg [162,163,164]. In another three RCTs of type 2 diabetic patients, all subjects receiving pramlintide also experienced modest weight loss, 0.5–2.6 kg [165,166,167]. These apparently meager findings are perhaps more remarkable considering that these subjects did not receive lifestyle modification therapy. Furthermore, traditional diabetic pharmacotherapy induces weight gain.

Pramlintide’s side effect profile was favorable, with adverse reactions limited to mild to moderate nausea and headache along with pain at the injection site [162,163,164,165,166,167,168]. Two recent studies investigating the efficacy of continuous infusion of pramlintide and insulin subcutaneously via a double pump system found this method of treatment to be an effective therapeutic option for patients who have difficulty maintaining optimal glycemic control and/or body weight [169,170].

The results described above suggest that amylin may also be an effective adjunct in the treatment of obesity. However, similar to the hyperleptinemia frequently seen in obese patients, obese individuals are frequently hyperamylinemic [145], which may suggest the development of long-term resistance to amylin’s effects, thus limiting the long-term efficacy of amylin treatment. As a result, long-term clinical trials investigating amylin treatment will be required to determine the clinical utility of amylin for the treatment of obesity.

### 4.8. Leptin

In 1950, Jackson Laboratories unintentionally identified a strain of mice that developed severe obesity early in life. These mice, which grew to be four times the size of their littermates, were termed ob/ob mice [171]. In 1958, a second strain of obese mutant mouse was discovered. In addition to their morbid obesity, these mice also developed severe, life-shortening diabetes, and so became known as the db/db mouse [172]. Later, it was discovered that these obese phenotypes were caused by mutations to the genes encoding leptin and the leptin receptor, respectively [173].

Leptin is an anorexigenic hormone predominantly derived from adipose tissue, found normally in the circulating plasma of mice and humans [171,174]. As with other anorexigenic hormones, the concentration of leptin in the blood fluctuates with feeding, increasing postprandially to limit further caloric intake [175,176]. Indeed, IV administration of leptin for 2 weeks to ob/ob mice decreased food intake, increased energy expenditure, and resulted in a 30% reduction in body weight, thus demonstrating its importance in bodyweight regulation [174,177]. Further, human congenital leptin deficiency is associated with early-onset obesity, which can be effectively treated with leptin-replacement therapy [178,179].

Leptin suppresses appetite by binding to the leptin receptor present on the surface of a subset of neurons in the hypothalamus and is a member of the gp130 family of cytokine receptors [180]. Specifically, leptin signaling in the ARC appears to be the primary mediator of its anorexigenic response. Leptin receptor is expressed in both NPY/AgRP and POMC/CART neurons in the ARC [181,182], and the reduced feeding induced by ICV administration of leptin is eliminated in rats with damaged ARC [183]. Upon binding its cognate ligand, the leptin receptor activates an associated janus kinase (JAK2) [184], and the resultant activation of signal transducer and activator of transcription-3 (STAT3) ultimately increases POMC transcription while suppressing transcription of AGRP [185]. These neurologic events help to explain leptin’s anorexigenic effects.

Leptin receptor-associated Jak2 also activates the phosphoinositide 3-kinase (PI3K) pathway via phosphorylation of insulin receptor substrate-2, (IRS-2), leading to the depolarization and activation of anorexigenic POMC-expressing neurons. This signaling cascade results in the downstream inactivation of transcription factor forkhead box O1 (FOXO1), leading to reduced expression of NPY and AgRP and increased POMC expression [186]. Additionally, leptin receptor signaling in multiple other areas of the hypothalamus inhibits 5′-AMP-activated protein kinase (AMPK), an energy sensing protein that is active during low energy states and stimulates feeding [186]. These myriad effects of leptin are believed to all work in concert and are key endogenous events in the induction of post-prandial satiety.

Based on these findings, the use of exogenous leptin as an anti-obesity therapy was initially viewed as a promising therapeutic option, and was investigated in multiple clinical trials. Unfortunately, obese patients express higher levels of leptin than their normal weight counterparts, resulting in the development of leptin tolerance over time. Because of this, clinical trials of leptin therapy in obese patients have thus far been disappointing [187]. Metreleptin is a leptin analog developed by Bristol-Myers Squibb/AstraZeneca. In a RCT, administration of leptin analog metreleptin for 16 weeks in 77 obese patients resulted in no body weight changes [188]. Another RCT with 30 patients also failed to demonstrate any significant placebo-adjusted weight loss in patients advised to maintain a hypocaloric diet [189]. As with many endogenous hormones with mild adverse event profiles, the adverse effects of metreleptin are limited to injection site reactions [188,189]. Currently, metreleptin is only indicated in lipodystrophy and not type 2 diabetes or obesity.

Despite the dismal clinical data for leptin in the treatment of obesity, recent work has renewed interest in leptin’s clinical translation. These studies provided a link between obesity-induced hypothalamic ER stress and leptin resistance [187,188]. Remarkably, administration of leptin in ob/ob, db/db, and diet-induced obese mice co-treated with chemical chaperones that relieve ER stress (4-phenyl butyric acid (PBA) and tauroursodeoxycholic acid (TUDCA)), resulted in increased leptin sensitivity and weight loss [187,188]. Future studies are needed to determine whether targeting this pathway will improve leptin’s viability as a weight loss drug.

#### Combining Leptin and Amylin

While leptin monotherapy for treatment of obesity is plagued by acquired resistance in humans and animals, preclinical studies have shown that the combination of leptin and amylin reduced weight to a greater extent than amylin alone. Moreover, obese rats pretreated with amylin display increased leptin signaling in the area postrema and VMN, suggesting that amylin restores leptin sensitivity in specific brain areas [190]. These findings, coupled with the fact that co-administration of leptin and other anorexigenic peptides (e.g., PYY and GLP-1/exendin-4 analog) does not increase their efficacy, suggests that amylin and leptin signaling play a specific synergistic role that may be exploited via therapeutics.

This hypothesis was evaluated clinically, and in a 24-week RCT, 177 overweight and obese individuals followed a 40% calorie-deficit diet and were treated with pramlintide during a 4-week lead-in period, with the target to achieve a 2%–8% body weight loss. Following the lead-in period, subjects who achieved the targeted weight loss were randomized to receive pramlintide plus metreleptin, pramlintide plus placebo, or metreleptin plus placebo. During this 20-week treatment period, subjects receiving a single drug exhibited a decline in the efficacy of the drug treatment, and eventually reached a weight plateau. However, subjects receiving combination therapy continued to lose weight, achieving a mean weight loss of 12.7% ± 0.9% (11.5 ± 0.9 kg), and, importantly, there was no weight loss plateau during the entire treatment period [191].

In a subsequent 28-week phase II trial, overweight and obese individuals treated with pramlintide/metreleptin lost an average of 11% of their baseline weight, significantly greater than placebo [192]. An extension of this 28-week trial demonstrated that subjects receiving pramlintide/metreleptin showed sustained weight loss, while those subjects receiving placebo regained almost all their weight [192]. The most common adverse effect associated with the pramlintide/metreleptin treatment was nausea [191,192].

Despite these promising results, development of pramlintide/metreleptin combination therapy was discontinued prior to phase III trials [193]. Like other hormone-replacement therapies, the necessity for frequent injections made this treatment modality impractical.

### 4.9. Oleoyl-Estrone

Oleoyl-estrone (OE) is an endogenous hormone derived from adipose tissue [194,195]. It circulates in the blood carried on HDL particles, and has been shown to reduce food intake while maintaining energy expenditure [194,195]. In obese rats, oral administration of OE helped to maintain appropriate glycemic control by increasing insulin sensitivity [196] and decreasing peripheral glucose utilization [197], though its exact mechanism of action is unknown [198]. Similar to leptin, oleoyl-estrone levels correlate with adiposity in humans, but obese individuals have lower circulating OE levels than would be expected by this model [195], perhaps suggesting that resistance to this hormone does not develop in the context of obesity.

A 14-day trial of IV administration of oleoyl-estrone to lean rats decreased food intake and induced dose-dependent weight loss [199], an effect that was maintained for 26 days after the conclusion of treatment [200]. Another preclinical study demonstrated that OE treatment preserved body protein by inducing selective targeting of fat stores for energy [201]. Additionally, unlike most anti-obesity therapies, OE can be administered orally, enhancing its therapeutic appeal. Unfortunately, Adán *et al*. found that, unlike lean rats, obese rats regain weight immediately after conclusion of OE administration, a finding that has been linked to the deficient leptinergic system observed in obesity [200].

Anecdotally, a morbidly obese (51.9 kg/m^2^) individual was administered oral oleoyl-estrone without dietary restrictions over ten consecutive 21-day trial periods followed by 2-month recovery periods. This individual reported a weight loss of 38.5 kg (BMI: 40.5 kg/m^2^) over 27 months and no side effects [202]. Upon this finding, along with animal data, Manhattan Pharmaceuticals (now TG Therapeutics, New York, NY, USA) sponsored clinical testing of the effectiveness of oral oleoyl-estrone in combating obesity. However, this investigation was discontinued in 2007 because RCTs failed to demonstrate significant placebo-adjusted weight loss [203]. Currently, there are no clinical trials investigating oleoyl-estrone.

### 4.10. Uroguanylin and Guanylyl Cyclases

Cyclic GMP (cGMP) is an important cellular second messenger responsible for the regulation of a wide range of physiologic processes, including intestinal fluid homeostasis, phototransduction in the retina and smooth muscle relaxation [204]. cGMP is generated by a class of receptors known as guanylyl cyclases, which catalyze the production of cGMP upon ligand binding. These receptors have a wide anatomic distribution and range of physiologic functions [204].

A potential role for the cGMP signaling axis in energy balance was initially suggested in part by findings in guanylyl cyclase C (*Gucy2c*) knockout mice, which demonstrated that *Gucy2c^−/−^* mice weighed more than their wild-type litter mates [205]. Expression of this receptor, initially thought to be limited to the intestinal epithelium, was later discovered in ARC neurons of the hypothalamus, where it was shown to modulate feeding through its cognate ligand uroguanylin [205]. Uroguanylin is produced in the intestine and released into the circulation postprandially, mimicking the pattern of expression and endocrine secretion seen in other anorexigenic gut-brain endocrine axes [205]. Importantly, both IV and ICV administration of uroguanylin induced satiety and decreased food intake in wild type, but not *Gucy2c^−/−^* mice [205]. This work revealed a role for the uroguanylin-GUCY2C axis among other known mechanisms of gut-brain signaling.

In addition to central appetite regulation, cGMP has also been shown to modulate energy balance by influencing adipose tissue composition. Mammalian adipose tissue exists either as brown adipose tissue (BAT) or white adipose tissue (WAT), and each of these tissues exhibit unique physiology [206]. WAT is involved in energy storage, and contributes to the pathogenic endocrine milieu typically associated with obesity and its related sequelae. Conversely, BAT plays a role in catabolism and thermogenesis due in part to increased mitochondria and uncoupler proteins (proteins which dissipate metabolic potential energy into heat, increasing energy expenditure) [206]. It was once thought that adult humans (who suffer most with the disease and sequelae of obesity) do not have meaningful levels of brown adipose tissue. However, recent studies utilizing positron-emission tomographic and computed tomographic (PET-CT) serendipitously discovered BAT in adult humans [206]. Although the overall prevalence and relevance of these findings are unclear (the study showed only between 1% and 10% of adults were positive), they have resulted in renewed excitement regarding the implications of BAT for human diseases.

Thus, with WAT mediating energy storage and BAT potentiating energy expenditure, manipulation of the pathways responsible for differentiation of adipocyte precursors to BAT or WAT may induce weight loss by selectively enriching BAT [207]. Due to this potential therapeutic benefit of driving adipose tissue toward a BAT fate, significant work has gone into study of adipocyte differentiation [208]. These studies have demonstrated a key role for cGMP, which potentiates brown fat differentiation through upstream guanylyl cyclases and their cognate natriuretic peptide ligands [207]. These effects were recapitulated through both transgenic and pharmacologic manipulation to accumulate cGMP levels in adipocytes [209]. Interestingly, a recent study reported the presence of Gucy2c and one of its ligands, guanylin, in macrophages derived from adipose tissue. Expression of these macrophages expressing Gucy2c and guanylin was shown to be mechanistically linked to resistance to diet-induced obesity [210]. More work is required to understand these findings in the larger context of obesity, but there is still a preponderance of evidence to support the role of cGMP signaling in modulating adipose tissue dynamics and obesity.

To date, uroguanylin homologues have not been investigated clinically for the treatment of obesity. However, there are several factors which paint an optimistic portrait for potential future clinical applications. First, the above referenced mouse studies provide a proof of concept for further investigation into humans. Additionally, an exogenous GUCY2C ligand known as linaclotide (Linzess, Ironwood Pharmaceuticals, Forest Laboratories), has already been approved by the FDA for use in humans to treat irritable bowel syndrome with constipation [211,212]. Clinical trials of linaclotide have shown it to be exceedingly well tolerated, lowering the regulatory burden for investigating the role of GUCY2C signaling in treating obesity.

## 5. Conclusions

Obesity has developed into a worldwide health crisis, and novel therapeutic strategies are desperately needed to offset its alarming spread. Past options have demonstrated limited efficacy or unacceptable adverse effects, or are limited by their expense and risk. However, recent advances in the knowledge of the body’s natural regulation of appetite have opened the door to new therapeutic strategies. In particular, the study of endocrine pathways that facilitate communication between the gut and central nervous system has generated new possible therapeutic targets for the pharmacologic treatment of obesity (Table 1). Further understanding of the dysfunction of these pathways in disease or the effects of their exogenous manipulation may yield a novel method to combat obesity.

**Table 1:** Current state of pharmacotherapeutic options targeting gut-brain endocrine axes. This table summarizes the pipeline of pharmacologic agents that target various gut-brain endocrine axes related to satiety. All therapeutic agents are administered subcutaneously unless noted otherwise. ^#^ GLP-1R and glucagon-R co-agonist.

Hormone Analog/Targeted Receptor	Phase in Development				
	Preclinical	Phase I	Phase II	Phase III	FDA Approved
Glucagon-like peptide 1 Receptor agonists		Oral Semaglutide ZP2929 ^#^	TT-401 ^#^	Lixisenatide	Exenatide (2004) Liraglutide (2010) Exenatide-LAR (2012)
MC4 Receptor agonists	AZD2820	LY2112688	RM-493		
Amylin					Pramlintide (2005)
Leptin				Metreleptin	
Oleoyl-Estrone			Oleoyl-estrone		
Ghrelin Inhibitors	NOX-B11 Ghrelin Antagonists GOAT Inhibitors		Ghrelin Vaccine (CYT009-GhrQb)		
Guanylyl Cyclase C agonists	Uroguanylin				Linaclotide (2012)
